# Deceased Donors With HIV in the Era of the HOPE Act: Referrals and Procurement

**DOI:** 10.1097/TXD.0000000000001641

**Published:** 2024-05-16

**Authors:** Tao Liang, Jordan H. Salas, Mary G. Bowring, Oyinkan Kusemiju, Brittany Barnaba, Matthew Wingler, Deborah McRann, Alghidak Salama, R. Patrick Wood, Allan Massie, William Werbel, Aaron A. R. Tobian, Dorry L. Segev, Christine M. Durand

**Affiliations:** 1 Department of Medicine, Johns Hopkins University School of Medicine, Baltimore, MD.; 2 Oregon Health & Science University School of Medicine, Portland, OR.; 3 Clinical Resource Solutions, LLC, Quincy, IN.; 4 Infinite Legacy, Baltimore, MD.; 5 Department of Surgery, Miller School of Medicine, University of Miami, Miami, FL.; 6 LifeGift Organ Procurement Organization, Houston, TX.; 7 Department of Surgery, New York University Grossman School of Medicine, New York, NY.; 8 Department of Pathology, Johns Hopkins University School of Medicine, Baltimore, MD.

## Abstract

**Background.:**

The HIV Organ Policy Equity Act legalizes organ procurement from donors with HIV (HIV D+). A prior survey of Organ Procurement Organizations (OPOs) estimated >2000 HIV D+ referrals/year; however, only 30–35 HIV D+/year have had organs procured. Given this gap, we sought to understand HIV D+ referrals and procurements in practice.

**Methods.:**

We prospectively collected data on all OPO-reported HIV D+ referrals, including reasons for nonprocurement. We evaluated trends and compared HIV D+ characteristics by procurement status using regression, chi-squared tests, and Wilcoxon rank-sum tests.

**Results.:**

From December 23, 2015 to May 31, 2021, there were 710 HIV D+ referrals from 49 OPOs, of which 171 (24%) had organs procured. HIV D+ referrals increased from 7 to 15 per month (*P* < 0.001), and the procurement rate increased from 10% to 39% (*P* < 0.001). Compared with HIV D+ without procurement, HIV D+ with procurement were younger (median age 36 versus 50 y), more commonly White (46% versus 36%), and more often had trauma-related deaths (29% versus 8%) (all *P* < 0.001). Nonprocurement was attributed to medical reasons in 63% of cases, of which 36% were AIDS-defining infections and 64% were HIV-unrelated, commonly due to organ failure (36%), high neurologic function (31%), and cancer (14%). Nonprocurement was attributed to nonmedical reasons in 26% of cases, commonly due to no authorization (42%), no waitlist candidates (21%), or no transplant center interest (20%).

**Conclusions.:**

In the early years of the HIV Organ Policy Equity Act, actual HIV D+ referrals were much lower than prior estimates; however, the numbers and procurement rates increased over time. Nonprocurement was attributed to both medical and nonmedical issues, and addressing these issues could increase organ availability.

The HIV Organ Policy Equity (HOPE) Act allows for transplantation using organs from donors with HIV (HIV D+) for recipients with HIV (HIV D+/R+).^1^ In practice, there have been about 35 HIV D+ with organs procured for transplant per year,^2^ falling far short of the projected potential, estimated at 300–600 per year.^3-5^ The reason for this gap remains unclear.

For donors without HIV, only a small percentage of donor referrals received by Organ Procurement Organizations (OPOs) ultimately result in organ procurement and transplantation. Procurement rates are difficult to quantify due to a lack of national, publicly available OPO data.^6^ A recent study including >100 000 referrals from 10 OPOs reported that only 5.1% resulted in organ procurement.^7^ For HIV D+ referrals in particular, data are limited. Cash et al^8^ performed a survey of all 58 OPOs that estimated >2000 HIV D+ referrals/year nationally. This survey predated HOPE Act implementation. Furthermore, it did not include whether HIV D+ referrals were suitable for organ procurement. Concerns have been raised that HIV D+ may have substantial medical contraindications to donation due to comorbidities or AIDS-defining illnesses.^4,5^

To better understand the potential of deceased HIV D+, we prospectively quantified and characterized OPO-reported HIV D+ referrals, procurement rates, and reasons for nonprocurement in the era of HOPE implementation.

## METHODS

### Study Design

Starting October 1, 2015, we began collecting information from OPOs on HIV D+ referrals (Johns Hopkins University Institutional Review Board, study number IRB00041681). In practice, HIV D+ organs could be procured for transplant on December 23, 2015 when the first HOPE in Action Multicenter Pilot Trial of HIV D+/R+ Kidney and Liver Transplantation opened (NCT02602262). To study procurement rates, the study period was December 23, 2015, to May 13, 2021.

All 58 OPOs were instructed to contact the study team in real time with any HIV D+ referrals, regardless of whether the OPO believed that the organs would be procured and utilized for transplant. The study team was available 24 h/d, 7 d/wk, initially by phone and, starting in March 2018, by pager. This HIV D+ referral study period spanned multiple HOPE in Action HIV D+/R+ Kidney and Liver Transplantation Recipient Studies (NCT02602262, NCT03734393, NCT03500315), conducted at 35 transplant centers in the United States (**Table S1**, **SDC**, http://links.lww.com/TXD/A654).

### Data Elements

We collected HIV D+ referral characteristics, including date of referral, specific OPO, Organ Procurement Transplantation Network (OPTN) region, donor age, sex, race, cause of death, mechanism of death, procurement status, and, when applicable, the reasons why organs were not procured and/or utilized.

The reasons for nonprocurement were grouped into several categories. If the donor was suspected or confirmed to have an AIDS-defining illness per Centers for Disease Control and Prevention definition, procurement was not allowed under the HOPE Act.^9^ This was considered an HIV-related medical contraindication and infection type was collected. Alternatively, OPOs could cite medical contraindications not related to HIV, which were further divided into (1) acute or chronic organ failure, (2) high neurologic function (ie, perceived low probability of progressing to circulatory death within a suitable timeframe for organ recovery), (3) non-AIDS defining infection/sepsis, (4) cancer, and (5) hepatitis C virus in donors >60 y old (guidance suggested by HOPE in Action transplant center investigators). Lastly, on March 27, 2020, (6) SARS-CoV-2 infection in the donor was added as a reason as organs from donors with SARS-CoV-2 were not being utilized for transplantation early in the COVID-19 pandemic. In cases where multiple medical contraindications were reported (3.2%), a physician (C.M.D.) and an OPO coordinator on the study team (M.W.) adjudicated the primary reason. For example, if both cancer and acute/chronic organ failure were selected, cancer would be considered the primary cause, as this is a more absolute contraindication to donation. Nonmedical reasons that could be cited included authorization issues (ie, unregistered donor and next-of-kin did not authorize donation), no candidates listing as willing to accept HIV D+ on the match list, no interest from transplant centers, OPO concerns about HIV disclosure, and OPO decision not to pursue without other criteria met.

### Statistical Analysis

All statistical analyses were conducted using Stata 18 software (StataCorp, College Station, TX). Descriptive statistics of categorical variables were reported as frequencies and percentages, and continuous variables were reported as medians and interquartile range. Wilcoxon rank-sum and chi-square tests were performed, for categorical and continuous variables respectively, to compare HIV D+ referrals by procurement status. We used univariable linear regression to visualize temporal trends in referral and procurement over the study period, and to determine whether referral and procurement rates increased or decreased over time, using calendar months as the unit of time.

## RESULTS

### Referral Outcomes of Deceased Donor Referrals With HIV

**FIGURE 1.**
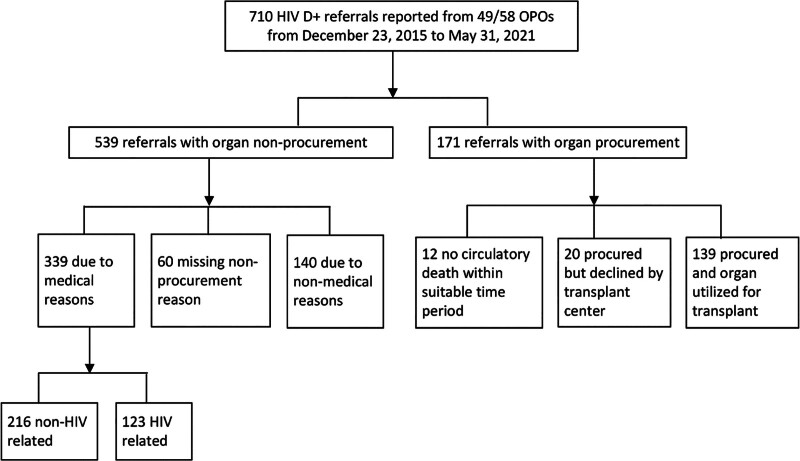
Referral outcomes of deceased HIV D+. HIV D+, donors with HIV; OPOs, Organ Procurement Organizations.

From December 23, 2015, to May 31, 2021, there were 710 HIV D+ referrals. Of these, 171 (24%) proceeded to procurement and 539 (76%) did not. Among those that proceeded to the operating room for procurement, 139 (81%) had organs utilized for transplant: 20 (12%) had organs procured but subsequently declined by transplant centers, and 12 (7%) did not experience circulatory death within a suitable timeframe for organ procurement (Figure 1).

Compared with HIV D+ with nonprocurement, HIV D+ with procurement were younger (median age 36 versus 50 y), more commonly White (46% versus 36%), more frequently had head trauma as a cause of death (29% versus 8%), and less frequently had a cardiovascular mechanism of death (15% versus 33%; all *P* < 0.001) (Table 1).

**TABLE 1.** Characteristics of HIV D+ referrals by procurement status

Characteristics	HIV D+ referrals with organ procurement, N = 171^*a*^	HIV D+ referrals with organ nonprocurement, N = 539^*b*^	*P * ^*c*^
Age, y, median (IQR)	36 (28–46)	50 (39–58)	<0.001
Male sex, n (%)	117 (69)	368 (71)	0.65
Race, n (%)			
White	79 (46)	173 (36)	<0.001
Black	58 (34)	277 (58)	
Other	33 (19)^*d*^	26 (5)^*e*^	
Cause of death, n (%)			
Anoxia	78 (46)	261 (63)	<0.001
Cerebrovascular/stroke	42 (25)	104 (25)	
Head trauma	50 (29)	33 (8)	
Other	0 (0)	15 (4)	
Mechanism of death, n (%)			
Cardiovascular	26 (15)	133 (33)	<0.001
Intracranial hemorrhage	46 (27)	90 (22)	
Drug intoxication	33 (19)	50 (12)	
Death from natural causes	6 (4)	49 (12)	
Blunt injury	35 (21)	20 (5)	
Other	24 (14)	58 (14)	

^*a*^Includes 44 donors with confirmed false-positive HIV tests; missingness for HIV D+ referral with procurement age (n = 1), sex (n = 1), race (n = 1), cause of death (n = 1), and mechanism of death (n = 1).

^*b*^Missingness for D+ referrals with nonprocurement: age (n = 5), sex (n = 31), race (n = 65), cause of death (n = 139), and mechanism of death (n = 152).

^*c*^Wilcoxon rank-sum *P* value for age; chi-square *P* values for sex and race; Fisher exact *P* values for cause of death and mechanism of death.

^*d*^Other races include Asian (n = 2), Native Hawaiian or other Pacific Islander (n = 1), multirace of White and Black (n = 1), multirace of White and unspecified race (n = 2), and unspecified race with Hispanic ethnicity (n = 27).

^*e*^Other races include Asian (n = 3), Native Hawaiian or other Pacific Islander (n = 2), American Indian or Alaska Native (n = 1), multirace of White and unspecified race (n = 1), unspecified race with Hispanic ethnicity (n = 14), unspecified race and ethnicity (n = 5).

HIV D+, donors with HIV; IQR, interquartile range.

### Trends Over Time

**FIGURE 2.**
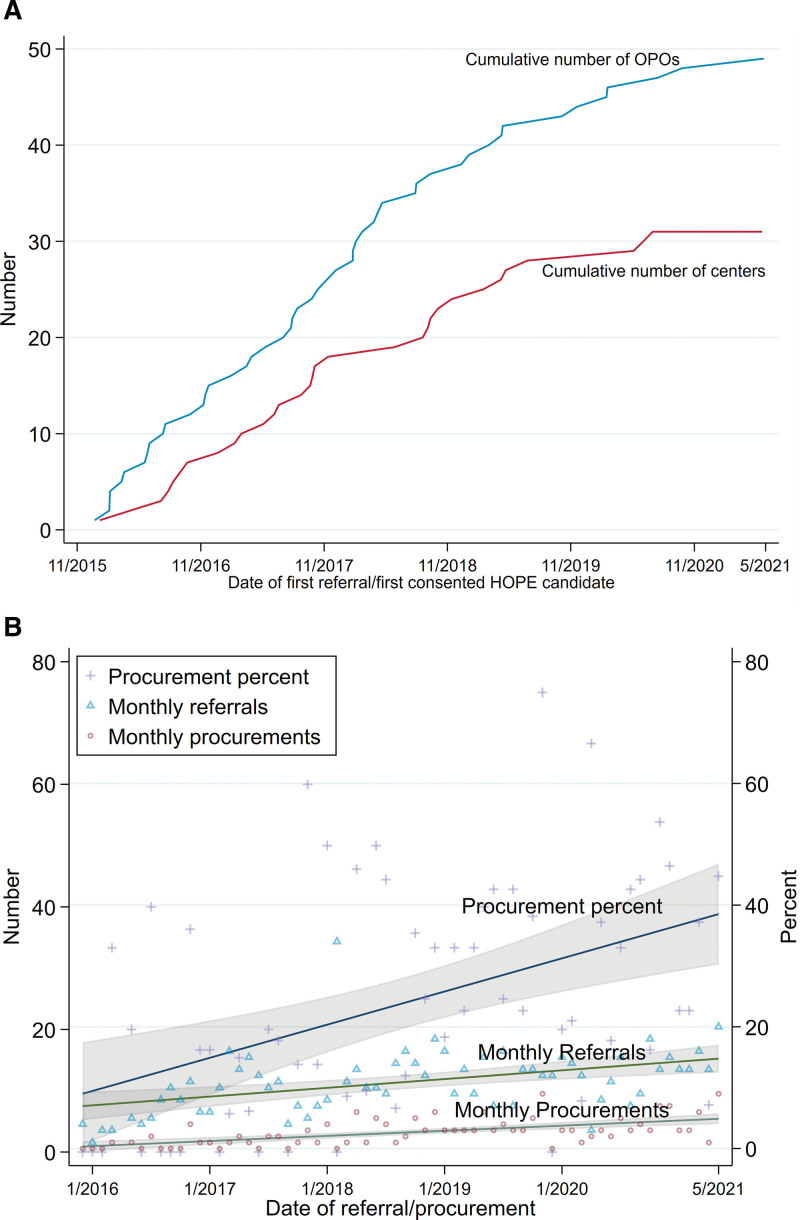
Trend over time in HOPE Act participation and HOPE donor procurement. Trends over time in (A) OPOs reporting HIV D+ referrals and transplant centers with consented candidates for HOPE trials. The cumulative number of OPOs that began reporting HIV D+ referrals is based on the first date of HIV D+ referral from that OPO. The cumulative number of transplant centers consenting candidates for HOPE is based on the first date a candidate was consented for HOPE at that center. B, HIV D+ referrals, procurements, and procurement percent. Each line represents the fitted univariable linear regression line of the displayed value over calendar months. HIV D+, donors with HIV; HOPE, HIV Organ Policy Equity; OPOs, Organ Procurement Organizations.

During the study period, the number of OPOs reporting at least 1 HIV D+ referral increased from 1 to 49 and the number of centers in our consortium consenting at least 1 candidate increased from 1 to 31 (Figure 2A). Over the same period, the number of HIV D+ referrals/month increased from 7 to 15 (*P* < 0.001), the number of procurements increased from 0.3 to 5 per month (*P* < 0.001), and the procurement rate increased from 10% to 39% (*P* < 0.001) (Figure 2B).

### Geographic Distribution of Deceased Donor Referrals With HIV

**FIGURE 3.**
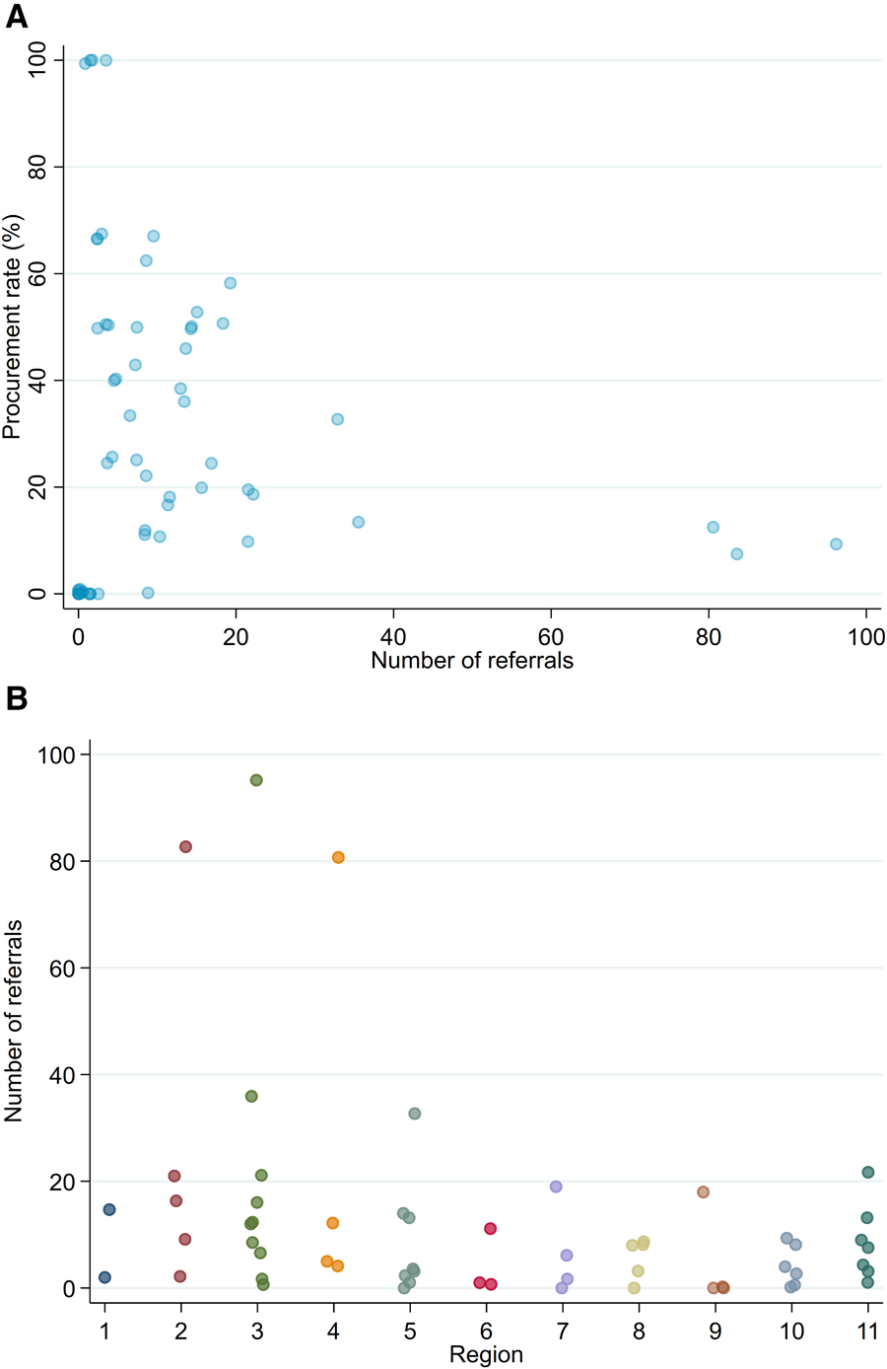
HOPE donor referrals and procurements by OPO. A, Overall procurement percent vs total HIV D+ referrals for each OPO and (B) total HIV D+ referrals for each OPO by OPTN region. Each point represents 1 OPO. Transparency and random jitter applied to illustrate overlap between OPOs. HIV D+, donors with HIV; OPO, Organ Procurement Organization; OPTN, Organ Procurement Transplantation Network.

Over the study period, 49 of 58 (84%) OPOs reported at least 1 HIV D+ referral and 44 of 58 (76%) had at least 1 HIV D+ referral with procurement (Figure 3A; **Table S2**, **SDC**, http://links.lww.com/TXD/A654). The median number of HIV D+ referrals was 9 (range, 0–96) and the median number of HIV D+ procurements was 2 (range, 0–11) (Figure 3A; **Table S2**, **SDC**, http://links.lww.com/TXD/A654). The median procurement rate was 33% (range, 0–100%). HIV D+ referrals were common from OPOs in OPTN regions 3 (30%), 2 (19%), and 4 (15%; Figure 3B; **Table S2**, **SDC**, http://links.lww.com/TXD/A654).

### Reasons for Nonprocurement of Organs

Among HIV D+ referrals with nonprocurement, 63% (339/539) were attributed to medical reasons, 26% (140/539) to nonmedical reasons, and 11% (60/539) did not have a reason cited (Figure 1). Among those with nonprocurement for medical reasons (Table 2), 64% (216/339) were unrelated to HIV and 36% (123/339) were due to suspected or confirmed AIDS-defining illness. Of these 123 cases, there were 148 infections cited, most commonly *Pneumocystis* pneumonia (53/148, 36%) and *Cryptococcus* meningitis (25/148, 17%) (Table 2). Among medical reasons for nonprocurement unrelated to HIV, 36% (77/216) were due to acute or chronic organ failure, 31% (67/216) were due to high neurologic function, and 14% (30/216) were due to cancer (Table 2).

**TABLE 2.** Reasons for organ nonprocurement among HIV D+

Reasons for nonprocurement	*n* (%)
Medical reasons not related to HIV	(N = 216)
Acute/chronic organ failure	77 (36)
High neurologic function	67 (31)
Cancer	30 (14)
Non-AIDS defining infection/sepsis (including CNS infections)	17 (8)
HCV and age >60	5 (2)
COVID-19	3 (1)
Other^*a*^	14 (6)
Unspecified	3 (1)
Medical reasons related to HIV, n (%)	(N = 148)^*b*^
*Pneumocystis* pneumonia	53 (36)
*Cryptococcus* meningitis	25 (17)
Toxoplasmosis	13 (9)
HIV-related encephalopathy	10 (7)
Nontuberculous mycobacteria	9 (6)
Cytomegalovirus disease	7 (5)
Kaposi’s sarcoma	6 (4)
Progressive multifocal leukoencephalopathy	4 (3)
Tuberculosis	4 (3)
Histoplasmosis	4 (3)
Esophageal candidiasis	4 (3)
HIV-related lymphoma	3 (2)
Coccidioidomycosis	3 (2)
HIV-related wasting syndrome	2 (1)
Chronic or disseminated herpes simplex	1 (<1)
Nonmedical reasons, n (%)	(N = 140)
Authorization issues	59 (42)
No candidates on the waitlist	29 (21)
Declined by transplant center	28 (20)
OPO decision not to pursue	17 (12)
OPO concerns about HIV disclosure	4 (3)
Other^*c*^	3 (2)

^*a*^Other included: organs too small (n = 1), overall clinical picture (n = 3), medical history (n = 1), donor expired before approach (n = 6), too unstable for donation (n = 2), and other, non-HIV related (n = 1).

^*b*^One hundred forty-eight AIDS-defining illnesses among 123 HIV D+ referrals.

^*c*^Other included: late referral (n = 2); coroner denied (n = 1).

CNS, central nervous system; HCV, hepatitis C virus; HIV D+, donors with HIV; OPO, Organ Procurement Organization.

Among those with nonmedical reasons for nonprocurement, 42% (59/140) were due to no authorization, 21% (29/140) were due to no waitlist candidates, and 19% (27/140) were due to no interest from transplant centers (28/140, 20%). Less commonly reported nonmedical reasons for nonprocurement included OPO not pursuing evaluation without other specific exclusion criteria (17/140, 12%) and OPO concerns regarding HIV disclosure (4/140, 3%).

## DISCUSSION

In this national prospective study of deceased donor referrals with HIV (HIV D+), 84% of OPOs across the US reported 710 HIV D+ referrals over a time period of 5.4 y. Of these, 24% proceeded to procurement; HIV D+ with procurement were younger and more likely to have a trauma-related death compared with HIV D+ with nonprocurement. The total number of HIV D+ referrals and procurement rates increased over time. The majority of HIV D+ referrals were from the northeastern and southern regions of the United States. Nonprocurement was attributed to medical reasons in 63% of cases, 36% of which were due to possible AIDS-defining infections, primarily *Pneumocystis* and *Cryptococcus*. Nonmedical reasons related to authorization, number of transplant candidates on the waitlist, or transplant center/OPO decision accounted for 26% of HIV D+ cases with nonprocurement.

Overall, we observed a much lower annual number of HIV D+ referrals in practice (131/y) compared with estimates of HIV D+ referrals from a prior OPO survey (2164/y).^8^ The OPO survey was based on retrospective records and/or OPO staff recollection before HOPE Act implementation, whereas our study was prospective and occurred when organs could truly be procured for transplantation. However, our study relied on OPOs voluntarily reporting all HIV D+ referrals, regardless of perceived procurement potential. Thus, it likely underestimated the total number of HIV D+ referrals. Notably, in both the survey and this prospective study, HIV D+ referrals were highest in the Southeast, which also aligns with the highest proportion of deaths among people with HIV in the United States, according to the Centers for Disease Control and Prevention.^10^

We also found far fewer HIV D+ referrals who proceeded to procurement (32/y) compared with estimates of suitable HIV D+ donors projected by prior studies (range, 356–652).^3-5^ The lower number in practice may be due to additional data on medical exclusions to donation (eg, granular data on neurologic function) or due to nonmedical exclusion factors, such as donor authorization status, that were not available in registry and retrospective studies of potential HIV D+. Of note, our observed 24% procurement rate for HIV D+ referrals is much higher than the 5% procurement rate for donors without HIV reported in a recent study of >100 000 referred donors.^7^ Our relatively high procurement rate might suggest that donor hospitals and/or OPOs were more likely to report HIV D+ with a high probability of procurement. Alternatively, this might suggest that transplant center acceptance rates for HIV D+ offers are high. A prior study of OPTN data from Wilk et al^11^ reported that the average organ acceptance rates across centers was higher for kidney and liver HIV D+ (47% and 60%, respectively) compared with donors without HIV (19% and 23%, respectively). Of note, we observed few HIV D+ referrals in which organs were procured but not utilized, occurring in only 4% of cases.

There were 123 HIV D+ who did not proceed to procurement because of a suspected or proven AIDS-defining illness. Notably, nearly half of these infections were *Pneumocystis* pneumonia or *Cryptococcus* meningitis. Since effective treatment is available for both infections, theoretically, organs could be used from these donors with appropriate prophylaxis in recipients (trimethoprim/sulfamethoxazole and fluconazole, respectively, which most recipients are already on as posttransplant prophylaxis).^12^ In practice, procurement is not currently allowed in these cases because a suspected AIDS-defining opportunistic infection is an absolute contraindication for HIV D+ under the HOPE Act Safeguards and Research Criteria.^9^ However, if HIV D+/R+ kidney and liver transplantation move from research to clinical care, as has been recommended by a federal advisory board,^13^ criteria for HOPE donors will be at the discretion of clinical transplant teams. If donor acceptance criteria are liberalized, it might increase incentives for OPOs to pursue these donors.

Nonmedical barriers were also cited as reasons for nonprocurement. Lack of authorization was stated in 10% of nonprocurement cases, which could be due to lack of donor registration or lack of authorization from a donor’s next-of-kin. A study by Nguyen et al^14^ showed low donor registration rates (21%) among people living with HIV, but a high willingness to donate (87%). Our study did not collect data on how often OPO staff approached next-of-kin for authorization in these HIV D+ referral cases. However, studies of referred donors without HIV have shown a high variability in OPO authorization approach rates.^7^

Other nonmedical issues such as concerns regarding HIV disclosure and OPO decision not to pursue accounted for closure in 4% of HIV D+ referrals. Perceived barriers and disincentives to pursue HIV D+ at the OPO level have been identified in prior studies.^15,16^ A study by Predmore et al,^16^ which included in-depth interviews with 20 OPO staff members, identified OPO concerns regarding HIV disclosure to next-of-kin and fear of HIV infection to procurement staff. In practice, these concerns should not be barriers to donation and procurement. HIV disclosure is not required to obtain authorization for donation. Moreover, standard universal precautions protect organ procurement staff from bloodborne transmissible diseases such as HIV.

Finally, a lack of waitlist candidates and no transplant center interest were cited in 17% of nonprocurement cases. This issue might be mitigated by broader transplant center participation in HIV D+/R+ transplantation. As of November 2022, only 31 transplant centers, roughly 13% of US transplant centers, had an OPTN variance to perform HIV D+/R+ transplants. Low transplant center participation might be due to current required experience thresholds under the HOPE Safeguards,^9,17^ or it might be due to the additional administrative effort and cost required for a research protocol. These barriers may be lowered if HIV D+/R+ transplantation moves from research to clinical care. Under current research criteria, only 3% of heart programs and 0% of lung programs are eligible to perform HIV D+/R+ transplants; if these criteria were expanded, 39% of both heart and lung programs could participate.^17^ In a study of OPO decisions to pursue HOPE donors, the number of potential organs that could be recovered was the most important attribute.^15^ Thus, the possibility of having multiple organs utilized from a donor would likely influence OPOs to evaluate and procure organs from more HIV D+ referrals.

Our study had several limitations. As previously acknowledged, we relied on voluntary reporting from OPO staff and it is unlikely that we received data on all HIV D+ referrals. However, we found that 84% of OPOS reported at least 1 HIV D+ referral. In some instances, our data were missing, and we could not ascertain the reason for nonprocurement. Moreover, medical contraindications were based on the judgment of the OPO staff, and we did not collect objective data such as laboratory values or neurologic exam data to support this decision. Finally, we did not have a control group of referred donors without HIV. Despite these limitations, this study provides insight into the current real-world decision-making process for HIV D+ referrals across the United States.

In summary, since the implementation of the HOPE Act, >130 HIV D+ referrals have been made each year, with the opportunity for a potentially life-saving transplant. In most cases, these organs were not procured, for medical and nonmedical reasons. Overall, referrals were much lower than prior survey estimates, although the numbers increased over time. These data highlight potential areas for intervention to expand and optimize organ procurement from HIV D+.

## Supplementary Material


