# Supplementary material for: Deceased Donors With HIV in the Era of the HOPE Act: Referrals and Procurement
Source: Transplant Direct. 2024 May 16;10(6):e1641. doi: 10.1097/TXD.0000000000001641 (PMC11104717; doi:10.1097/TXD.0000000000001641)
Supplement: Supplementary file 1 [file txd-10-e1641-s001.pdf]

Table S1. Transplant centers participating in HOPE in Action trials between December 23, 2015 to May 31, 2021.

Transplant Centers
CTYN – Yale New Haven Hospital
MAMG – Massachusetts General Hospital
DCGU – MedStar Georgetown Transplant Institute
MDJH – Johns Hopkins Hospital
MDUM – University of Maryland Medical System
NJSB – Saint Barnabas Medical Center
PAHM – Hahnemann University Hospital
PAPT – University of Pittsburgh Medical Center
PAUP – Hospital of the University of Pennsylvania
ALUA – University of Alabama Hospital
ARUA – UAMS Medical Center
FLCC – Cleveland Clinic Florida Weston
FLJM – Jackson Memorial Hospital University of Miami School of Medicine
GAEM – Emory University Hospital
LAOF – Ochsner Foundation Hospital
TXMC – Methodist Dallas Medical Center
TXSP – UT Southwestern Medical Center/William P. Clements Jr. University Hospital
CASD – University of California San Diego Medical Center
CASF – University of California San Francisco Medical Center
CAUC – University of California at Los Angeles Medical Center
ILNM – Northwestern Memorial Hospital
ILPL – Rush University Medical Center
ILUI – University of Illinois Medical Center
MNUM – University of Minnesota Medical Center
COUC – University of Colorado Hospital/Health Science Center
MOBH – Washington University in St. Louis/Barnes Jewish Hospital
NYCP – NY Presbyterian Hospital/Columbia University Medical center
NYMS – Mount Sinai Medical Center
NYNY – New York – Presbyterian Hospital/Weill Cornell Medical Center
NYUC – NYU Langone Health
INIM – Indiana university Health
OHUC – University of Cincinnati Medical Center
NCDU – Duke University Hospital
VAMC – VCU Health System Authority, VCUMC (VAMC)
VAUV – University of Virginia Health Sciences Center

Table S2. HIV D+ referrals by OPOs from December 23, 2015 to May 31, 2021

OPO	Total HIV D+ referrals	HIV D+ referrals with procurement	Procurement rate
**Overall** Median; IQR; range	9; (4, 15); [0, 96]	2; (1, 5);[0, 11]	33; (14, 50); [0, 100]
**Individual OPOS**			
OPO-1	96	9	9%
OPO-2	83	6	7%
OPO-3	81	10	12%
OPO-4	36	5	14%
OPO-5	33	11	33%
OPO-6	22	4	18%
OPO-7	21	4	19%
OPO-8	21	2	10%
OPO-9	19	11	58%
OPO-10	18	9	50%
OPO-11	17	4	24%
OPO-12	15	8	53%
OPO-13	15	3	20%
OPO-14	14	7	50%
OPO-15	14	7	50%
OPO-16	14	5	36%
OPO-17	13	6	46%
OPO-18	13	5	38%
OPO-19	12	2	17%
OPO-20	11	2	18%
OPO-21	10	1	10%
OPO-22	9	6	67%
OPO-23	9	2	22%
OPO-24	9	1	11%
OPO-25	9	0	0%
OPO-26	8	4	50%
OPO-27	8	5	63%
OPO-28	8	1	13%
OPO-29	8	2	25%
OPO-30	7	3	43%
OPO-31	6	2	33%
OPO-32	5	2	40%
OPO-33	5	2	40%
OPO-34	4	2	50%
OPO-35	4	1	25%

OPO-36	4	1	25%
OPO-37	4	2	50%
OPO-38	3	0	0%
OPO-39	3	2	67%
OPO-40	3	2	67%
OPO-41	3	3	100%
OPO-42	3	2	67%
OPO-43	2	1	50%
OPO-44	2	0	0%
OPO-45	2	2	100%
OPO-46	1	1	100%
OPO-47	1	0	0%
OPO-48	1	1	100%
OPO-49	1	0	0%
OPO-50	0	0	-
OPO-51	0	0	-
OPO-52	0	0	-
OPO-53	0	0	-
OPO-54	0	0	-
OPO-55	0	0	-
OPO-56	0	0	-
OPO-57	0	0	-
OPO-58	0	0	-

*OPOs are listed in the order of the number of referrals received
